# What Can Article-Level Metrics Do for You?

**DOI:** 10.1371/journal.pbio.1001687

**Published:** 2013-10-22

**Authors:** Martin Fenner

**Affiliations:** Article-Level Metrics Project, Public Library of Science, San Francisco, California, United States of America

## Abstract

Article-level metrics (ALMs) provide a wide range of metrics about the uptake of an individual journal article by the scientific community after publication. They include citations, usage statistics, discussions in online comments and social media, social bookmarking, and recommendations. In this essay, we describe why article-level metrics are an important extension of traditional citation-based journal metrics and provide a number of example from ALM data collected for *PLOS Biology*.

## Abstract 2

Martin Fenner discusses the PLOS article-level metrics and how they can be used to assess the value of published research as part of the Tenth Anniversary *PLOS Biology* Collection.

The scientific impact of a particular piece of research is reflected in how this work is taken up by the scientific community. The first systematic approach that was used to assess impact, based on the technology available at the time, was to track citations and aggregate them by journal. This strategy is not only no longer necessary—since now we can easily track citations for individual articles—but also, and more importantly, journal-based metrics are now considered a poor performance measure for individual articles [1],[2]. One major problem with journal-based metrics is the variation in citations per article, which means that a small percentage of articles can skew, and are responsible for, the majority of the journal-based citation impact factor, as shown by Campbell [1] for the 2004 *Nature* Journal Impact Factor. Figure 1 further illustrates this point, showing the wide distribution of citation counts between *PLOS Biology* research articles published in 2010. *PLOS Biology* research articles published in 2010 have been cited a median 19 times to date in Scopus, but 10% of them have been cited 50 or more times, and two articles [3],[4] more than 300 times. *PLOS Biology* metrics are used as examples throughout this essay, and the dataset is available in the supporting information (Data S1). Similar data are available for an increasing number of other publications and organizations.

**Figure 1:**
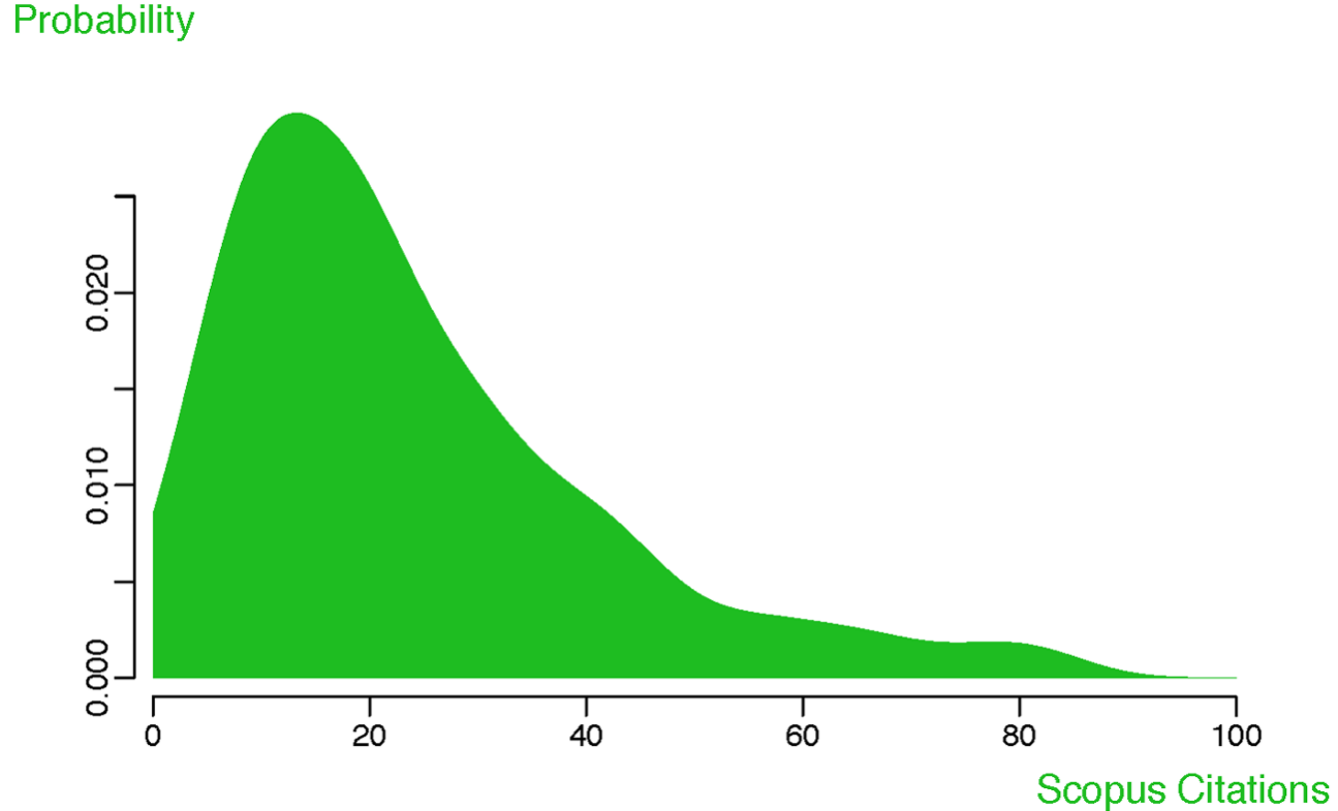
Citation counts for *PLOS Biology* articles published in 2010. Scopus citation counts plotted as a probability distribution for all 197 *PLOS Biology* research articles published in 2010. Data collected May 20, 2013. Median 19 citations; 10% of papers have at least 50 citations.

Scientific impact is a multi-dimensional construct that can not be adequately measured by any single indicator [2],[5],[6]. To this end, PLOS has collected and displayed a variety of metrics for all its articles since 2009. The array of different categorised article-level metrics (ALMs) used and provided by PLOS as of August 2013 are shown in Figure 2. In addition to citations and usage statistics, i.e., how often an article has been viewed and downloaded, PLOS also collects metrics about: how often an article has been saved in online reference managers, such as Mendeley; how often an article has been discussed in its comments section online, and also in science blogs or in social media; and how often an article has been recommended by other scientists. These additional metrics provide valuable information that we would miss if we only consider citations. Two important shortcomings of citation-based metrics are that (1) they take years to accumulate and (2) citation analysis is not always the best indicator of impact in more practical fields, such as clinical medicine [7]. Usage statistics often better reflect the impact of work in more practical fields, and they also sometimes better highlight articles of general interest (for example, the 2006 *PLOS Biology* article on the citation advantage of Open Access articles [8], one of the 10 most-viewed articles published in *PLOS Biology*).

**Figure 2:**
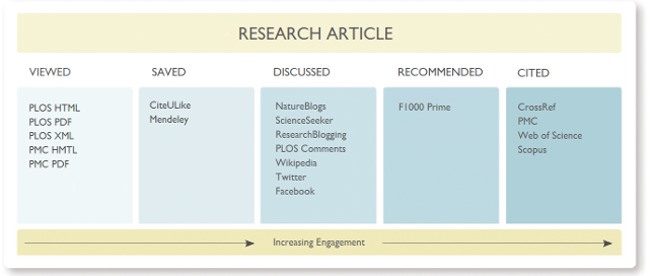
Article-level metrics used by PLOS in August 2013 and their categories. Taken from [10] with permission by the authors.

A bubble chart showing all 2010 *PLOS Biology* articles (Figure 3) gives a good overview of the year's views and citations, plus it shows the influence that the article type (as indicated by dot color) has on an article's performance as measured by these metrics. The weekly *PLOS Biology* publication schedule is reflected in this figure, with articles published on the same day present in a vertical line. Figure 3 also shows that the two most highly cited 2010 *PLOS Biology* research articles are also among the most viewed (indicated by the red arrows), but overall there isn't a strong correlation between citations and views. The most-viewed article published in 2010 in *PLOS Biology* is an essay on Darwinian selection in robots [9]. Detailed usage statistics also allow speculatulation about the different ways that readers access and make use of published literature; some articles are browsed or read online due to general interest while others that are downloaded (and perhaps also printed) may reflect the reader's intention to look at the data and results in detail and to return to the article more than once.

**Figure 3:**
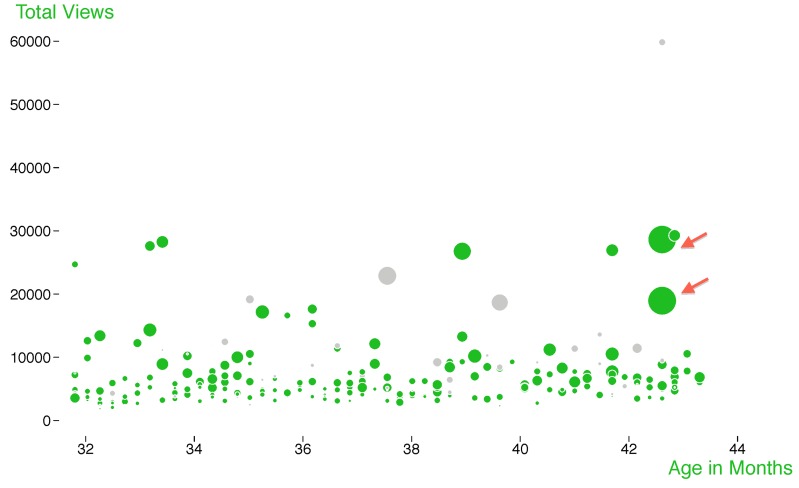
Views vs. citations for *PLOS Biology* articles published in 2010. All 304 *PLOS Biology* articles published in 2010. Bubble size correlates with number of Scopus citations. Research articles are labeled green; all other articles are grey. Red arrows indicate the two most highly cited papers. Data collected May 20, 2013.

When readers first see an interesting article, their response is often to view or download it. By contrast, a citation may be one of the last outcomes of their interest, occuring only about 1 in 300 times a PLOS paper is viewed online. A lot of things happen in between these potential responses, ranging from discussions in comments, social media, and blogs, to bookmarking, to linking from websites. These activities are usually subsumed under the term “altmetrics,” and their variety can be overwhelming. Therefore, it helps to group them together into categories, and several organizations, including PLOS, are using the category labels of Viewed, Cited, Saved, Discussed, and Recommended (Figures 2 and 4, see also [10]).

**Figure 4:**
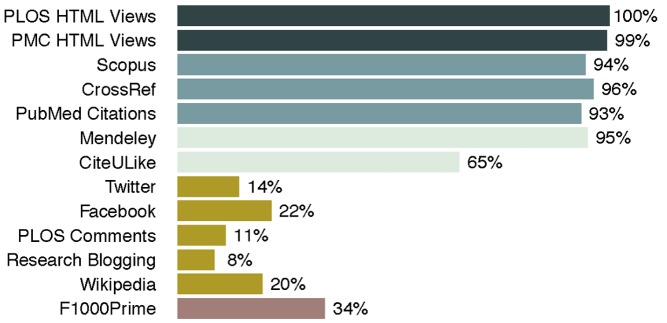
Article-level metrics for *PLOS Biology*. Proportion of all 1,706 *PLOS Biology* research articles published up to May 20, 2013 mentioned by particular article-level metrics source. Colors indicate categories (Viewed, Cited, Saved, Discussed, Recommended), as used on the PLOS website.

All *PLOS Biology* articles are viewed and downloaded, and almost all of them (all research articles and nearly all front matter) will be cited sooner or later. Almost all of them will also be bookmarked in online reference managers, such as Mendeley, but the percentage of articles that are discussed online is much smaller. Some of these percentages are time dependent; the use of social media discussion platforms, such as Twitter and Facebook for example, has increased in recent years (93% of *PLOS Biology* research articles published since June 2012 have been discussed on Twitter, and 63% mentioned on Facebook). These are the locations where most of the online discussion around published articles currently seems to take place; the percentage of papers with comments on the PLOS website or that have science blog posts written about them is much smaller. Not all of this online discussion is about research articles, and perhaps, not surprisingly, the most-tweeted PLOS article overall (with more than 1,100 tweets) is a *PLOS Biology* perspective on the use of social media for scientists [11].

Some metrics are not so much indicators of a broad online discussion, but rather focus on highlighting articles of particular interest. For example, science blogs allow a more detailed discussion of an article as compared to comments or tweets, and journals themselves sometimes choose to highlight a paper on their own blogs, allowing for a more digestible explanation of the science for the non-expert reader [12]. Coverage by other bloggers also serves the same purpose; a good example of this is one recent post on the OpenHelix Blog [13] that contains video footage of the second author of a 2010 *PLOS Biology* article [14] discussing the turkey genome.

F1000Prime, a commercial service of recommendations by expert scientists, was added to the PLOS Article-Level Metrics in August 2013. We now highlight on the PLOS website when any articles have received at least one recommendation within F1000Prime. We also monitor when an article has been cited within the widely used modern-day online encyclopedia, Wikipedia. A good example of the latter is the Tasmanian devil Wikipedia page [15] that links to a *PLOS Biology* research article published in 2010 [16]. While a F1000Prime recommendation is a strong endorsement from peer(s) in the scientific community, being included in a Wikipedia page is akin to making it into a textbook about the subject area and being read by a much wider audience that goes beyond the scientific community.

*PLOS Biology* is the PLOS journal with the highest percentage of articles recommended in F1000Prime and mentioned in Wikipedia, but there is only partial overlap between the two groups of articles because they focus on different audiences (Figure 5). These recommendations and mentions in turn show correlations with other metrics, but not simple ones; you can't assume, for example, that highly cited articles are more likely to be recommended by F1000Prime, so it will be interesting to monitor these trends now that we include this information.

**Figure 5:**
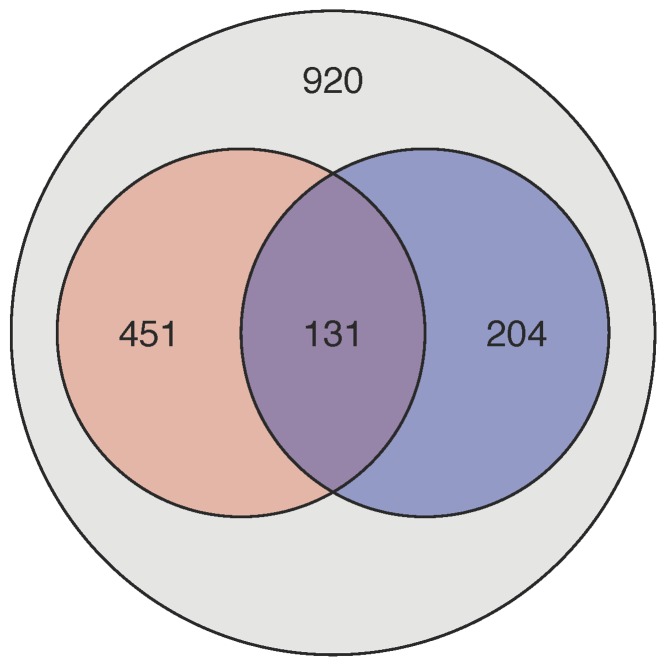
*PLOS Biology* articles: sites of recommendation and discussion. Number of *PLOS Biology* research articles published up to May 20, 2013 that have been recommended by F1000Prime (red) or mentioned in Wikipedia (blue).

With the increasing availability of ALM data, there comes a growing need to provide tools that will allow the community to interrogate them. A good first step for researchers, research administrators, and others interested in looking at the metrics of a larger set of PLOS articles is the recently launched ALM Reports tool [17]. There are also a growing number of service providers, including Altmetric.com [18], ImpactStory [19], and Plum Analytics [20] that provide similar services for articles from other publishers.

As article-level metrics become increasingly used by publishers, funders, universities, and researchers, one of the major challenges to overcome is ensuring that standards and best practices are widely adopted and understood. The National Information Standards Organization (NISO) was recently awarded a grant by the Alfred P. Sloan Foundation to work on this [21], and PLOS is actively involved in this project. We look forward to further developing our article-level metrics and to having them adopted by other publishers, which hopefully will pave the way to their wide incorporation into research and researcher assessments.

## Supporting Information

Data S1 **Dataset of ALM for** ***PLOS Biology*** **articles used in the text, and R scripts that were used to produce figures.** The data were collected on May 20, 2013 and include all *PLOS Biology* articles published up to that day. Data for F1000Prime were collected on August 15, 2013. All charts were produced with R version 3.0.0.
(ZIP)
